# Graded concentrations of digestible lysine on performance of White Leghorn laying hens fed sub-optimal levels of protein

**DOI:** 10.5713/ajas.20.0016

**Published:** 2020-05-12

**Authors:** Venkata Rama Rao Savaram, Shyam Sundar Paul, Venkata Lakshmi Narasimha Raju Mantina, Nagalakshmi Devanaboyina, Prakash Bhukya

**Affiliations:** 1 ICAR-Directorate of Poultry Research, Rajendranagar, Hyderabad-500030, Telangana, India; 2 College of Veterinary Science, PVNR Telangana Veterinary University, Hyderabad 500030, India

**Keywords:** Egg Mass, Egg Production, Egg Weight Feed Efficiency, Layer, Lysine

## Abstract

**Objective:**

An experiment was conducted to study the effect of graded concentration of digestible lysine (dLys) on performance of layers fed diets containing sub-optimal level of protein.

**Methods:**

Five diets were formulated to contain graded concentrations of dLys (0.700%, 0.665%, 0.630%, 0.593%, and 0.563%), but similar levels of crude protein (15% CP), energy (10.25 MJ ME/kg) and other nutrients. A total of 3,520 hens (26 wk of age) with mean body weight of 1,215+12.65 g were randomly divided into 40 replicate groups of 88 birds in each and housed in an open sided colony cage house. Each diet was offered *ad libitum* to eight replicates from 27 to 74 wk of age. The performance was compiled at every 28 d and the data for each parameter were grouped into three phases, that is early laying phase (27 to 38 wk), mid laying phase (39 to 58 wk), and late laying phase (59 to 74 wk of age) for statistical analysis.

**Results:**

Egg production, egg mass and feed efficiency (feed required to produce an egg) were significantly improved by the dLys level during the early and mid laying phases but not during the late phase. Whereas feed intake was significantly reduced by dLys concentration during mid and late laying phases but not during early laying phase. The egg weight was not affected by dLys concentration in any of the three phases.

**Conclusion:**

Based on best fitted statistical models, dietary requirements of dLys worked out to be 0.685%, 0.640%, and 0.586% during early phase, mid phase, and late egg laying phase, respectively. The calculated requirement of dLys for the respective production phases are 727 mg/b/d during the early and mid laying phases and 684 mg/b/d during the late laying phase in diets containing 15% CP.

## INTRODUCTION

Utilization of dietary protein is higher at sub-optimal concentration of feeding and thus the excretion of nitrogen is proportionately less at lower dietary concentrations of crude protein (CP) [1]. Hence, with increased availability of synthetic amino acids (AA) at reasonable price, diet formulation with precise concentrations of AA and minimum level of protein were adopted to reduce cost and nitrogen excretion [2]. The AA requirements of laying hens are generally based on performance criteria. During past two decades, productivity, persistency of lay and feed efficiency (FE) of laying hens increased substantially while body weight (BW) has decreased. Therefore, the NRC [3] recommendations may not be accurate in predicting requirements of AA for current genotypes of laying hen. Most AA requirement studies on layers have been conducted during early [4,5] or late production [6,7] phases. Experiments conducted for short duration or part of production cycle may not truly represent the entire production cycle. Meluzzi et al [1] found that low CP (13%) diet supplemented with AA sustained performance during the initial 8 wk of the experiment, after which EP and egg mass (EM) decreased compared to those fed diets with the recommended CP level (17%).

Establishment of precise AA requirements for different ages would allow full expression of the genetic potential throughout their laying phases besides creation of more precise, environment friendly and economical feeding program. The present experiment was conducted on White Leghorn (WL) layers of Babcock strain for almost full laying cycle i.e. from 27 to 74 wk of age, to evaluate the effects of graded concentrations of digestible lysine (dLys) in low CP diet on the production performance and to determine their optimum dietary concentrations under tropical condition.

## MATERIALS AND METHODS

### Birds and management

#### Birds

The experiment was conducted by following the guidelines of the Institute Animal Ethical Committee (approval number IAEC/DPR/6/2015). A total of 3,520 commercial layers (26 wk of age) of Babcock strain with mean BW of 1,215+12.65 g were placed in 4 bird colony cages and 22 adjacent colony cages having a common feeder were considered as a replicate. Thus, all the birds were equally housed in 40 replicate groups. Eight replicates (88 birds in each) were assigned to each of the five treatments in a completely randomized design. The cages were placed in an open-sided poultry house fitted on an elevated platform. Fluorescent bulbs were used to provide 16 h light period daily, including the daylight. The average minimum and maximum temperatures in the house during the three production phases experiment were 23.2°C±3.85°C and 37.5°C±6.26°C, respectively.

#### Diets

Five diets were formulated to contain graded concentrations of dLys (0.700%, 0.665%, 0.630%, 0.593%, and 0.563%), but similar levels of protein (15% CP), energy (10.25 MJ ME/kg) and other nutrients. Diets were prepared utilizing maize, soybean meal, sunflower cake, rapeseed cake, and deoiled rice bran to meet the recommended nutrient levels as per the feeding standards of the strain used [8] except for protein and AA. All the feed ingredients were analysed [9] for total Lys (Lys), methionine (Met), threonine (Thr), and tryptophan (Try) at the beginning of the experiment, and also as and when a new consignment of feed ingredient was received for feed compounding. The digestible coefficients for the above AA as suggested by Evonik (SEA) PTE LTD (Asia South Ingredient Report 2017) for the respective ingredients were used to calculate the concentrations of digestible Lys (dLys), methionine (dMet), threonine (dThr), and tryptophan (dTry) in diets. The levels of maize, soybean meal, sunflower meal, DL-Met and L-Lys HCl were altered to achieve the desired concentration of AA in experimental diets. Each diet was offered *ad libitum* to eight replicates (88 birds/replicate) from 27 to 74 wk of age.

#### Traits measured

Egg production (EP) was recorded twice daily from 27 to 74 wk of age. Feed intake (FI) and the quantity of feed consumed to produce a unit weight of egg (FE) were compiled at 28 d intervals (period). The average egg weight (EW) was recorded by weighing 60 randomly selected eggs per replicate during the last 3 days of each period. The EM was calculated by multiplying the average EW with the total number of eggs produced in each replicate and expressed as g per hen per period. The BW of the birds was recorded at the beginning (27 wk of age) and at the end of the experiment (74 wk of age). Number of birds died during experiment was recorded to calculate the livability.

### Statistical analysis

The data for each parameter were grouped into three phases and statistical analysis was carried out separately for each of the three phases. Data were analysed by the method of analysis of contrast variables using the general linear model procedures on analysis of variance for repeated measures using the Greenhouse-Geisser adjusted univariate significance tests as described by Littell et al [10] using SPSS [11]. Differences were considered to be significant at p<0.05, whereas a trend was considered to exist if P was between 0.05 to 0.10. Sum of squares for the treatment effect due to dLys concentration was further separated using orthogonal contrasts into single degree of freedom comparisons that included linear, quadratic, cubic and order 4 components of the response to the dLys concentration. Contrast analysis using Helmert contrast (which compares each level except the last with mean of all subsequent concentration) was also carried out to compare concentration of dLys to find out plateau, if any. For response variables with no significant effect of dLys concentration, the lowest level of dLys used in the study was considered as adequate. For response variables with significant effect of dLys concentration, four mathematical models were used to estimate the optimal level of dLys inclusion in diets to maximize layer responses based on a gradient treatment structure [12]. The EP, EW, FI, and FE were selected as layer responses. Linear broken-line, quadratic broken line (QBL), and exponential models were estimated using a nonlinear procedure, whereas the quadratic polynomial (QP) regression model was estimated using a regression procedure [11]. Details of the statistical models used were as described earlier [13]. The dLys requirements estimates based on 95% of maximal response were considered as optimum requirement level. A model with highest coefficient of determination values and smallest sum of square of residuals was considered as the best fit model. Practical dLys requirements were calculated by averaging estimates across four variables (EP, FE, EW, and FI) as per the strategy adopted earlier [13]. Estimates of requirements of digestible sulfur AA (dSAA) were derived from dLys requirement estimates based on the actual average ratio of these AA to that of dLys in the present study.

## RESULTS

Data on ingredient and nutrient composition of experimental diets has been presented in Table 1. ME, calcium and available P contents in diet of all the groups were comparable. Protein content of all the diets was about 15% which is considered adequate to meet requirement of nonessential AA (NRC [3]). The concentrations of dLys and dMet in soybean meal, sunflower cake, mustard cake, deoiled rice bran and maize were respectively 2.655% and 1.156%; 0.451% and 0.937%; 1.621% and 1.412%; 0.727% and 0.716%; 0.210% and 0.330%. The diets contained graded concentration of limiting AA (LAA) in fixed ratio to dLys. The average digestible LAA ratio during the entire study was dMet 51.6%, dSAA 87.9%, dThr 75.5%, and dThr 24.2% relative to dLys. Dietary variation in dLys concentrations did not influence (p<0.05) the final BW of layers (Av. BW at 74th wk were 1,581, 1,575, 1,586, 1,574, and 1,575 g in groups fed 0.700%, 0.665%, 0.630%, 0.593%, and 0.563% dLys, respectively.

### Early laying

EP and EM were significantly (p<0.05) increased by the increase in concentrations of dLys in diet (Table 2) and also varied between periods, but interaction between period and dLys was non-significant (Table 2). Increasing the concentration of dLys showed linear response on EP and EM. Helmert contrast of EP and EM data indicated that EP and EM varied significantly among periods with peak at period 2 (31 to 34 wk of age).

The FI was not significantly affected by dLys concentration but varied significantly among periods. FE was significantly (p<0.01) improved with the concentrations of dLys in diet and the periods (Table 2). Increasing the level of dLys showed quadratic effect on FE.

The EW was not affected by the variation in concentrations of dLys in diet (Table 2) but was influenced significantly by period. Helmert contrast of EW data indicated significant variation among periods.

These analyses indicated that the lowest dietary level of dLys used in the study (0.563% in diet) was adequate for optimal FI and EW responses. The dLys requirement estimates based on 100% and 95% of optimal responses for EP and FE were calculated from the mathematical model providing the best fit to the data set and have been shown in Table 6. In phase 1, for EP the best model (Exponential model) estimated dLys requirement (adequate for 95% of optimal response) at 0.815%; for EM the best model (QBL) estimate was 0.660%; for FE the best model (QP model) estimate was 0.581%. On averaging values across responses where mathematical models were statistically significant (EP, EM, and FE), the dLys requirement (% in diet) value during phase 1 works out to be 0.685%.

### Mid laying

EP and EM were significantly (p<0.05) increased with the concentrations of dLys in diet and also varied between periods, but interaction between period and dLys was non-significant (Table 3). Increasing the concentration of dLys showed significant quadratic response on EP and EM. Helmert contrast of EP or EM data indicated that EP or EM varied significantly among periods.

The FI was significantly (p<0.01) reduced by dLys concentration and also the periods. Increasing the concentration of dLys showed significant order 4 response on FI.

The FE was significantly (p<0.01) improved by the variation in concentrations of dLys in diet and also the periods (Table 4) but interaction between period and dLys concentration was non-significant. EW was not affected by the variation in concentrations of dLys in diet (Table 4) but was increased significantly with the age of the bird. Helmert contrast of EW data indicated significant variation among periods, except between period 4 and 5. The EW continued to increase with age of hen throughout the experiment.

These analyses indicated that the lowest dietary level of dLys used in the study (0.563% in diet) was adequate for optimal EW response. The dLys requirement estimates based on dLys intake for 100% and 95% of optimal responses for EP, FE, and FI were calculated from mathematical model providing best fit to the dataset and have been shown in Table 6. In phase 2, the best model (QP model) estimated dLys requirement (95% of optimal response) at 0.612%; for EP, 0.665% for EM, 0.712% for FE, and 0.573% for FI. On averaging values across responses where mathematical models were statistically significant (EP, EM, FE, and FI), the dLys requirement for phase 2 has been worked out to be 0.640%.

### Late laying

EP and EM were not significantly (p>0.05) affected by the variation in concentrations of dLys in diet (Table 5). The EP was significantly (p<0.01) reduced with the age, but interaction between period and dLys was non-significant (Table 5). Helmert contrast of EP or EM data indicated EP or EM varied significantly among periods with a gradual declining trend with each passing period.

The FI was significantly (p<0.05) and quadratically reduced by dLys concentration and varied significantly among periods. FE was not significantly (p>0.05) affected by the variation in concentrations of dLys in diet but varied among periods (Table 5). Increasing the concentration of dLys showed order 4 effect on FE.

The EW was not affected by the variation in concentrations of dLys in diet (Table 6) but was significantly increased with advancement of the bird age. Helmert contrast of EW data indicated significant variation among periods and indicated a gradual increase in EW with increasing age.

These analyses indicated that the lowest dietary level of dLys used in the study (0.563%) was adequate for optimal EP, FE, and EW responses. The dLys requirement estimates based on 100% and 95% of optimal responses in FI were calculated from the best fitted mathematical model and have been shown in Table 5. In phase 3, for FI, the best model (exponential model) estimated dLys requirement (95% of optimal response) at 0.586%. On averaging values across responses where mathematical models were statistically significant (FI), the dLys requirement (% in diet) values for phase 3 works out to be 0.586%.

## DISCUSSION

Among the AA requirements, Lys is especially important because it is used as the basis for setting the requirements for all other AA [2,14]. Multiple factors like basal diet, genetic lines, ambient temperature, age, carry over effect of previous concentration of AAs, etc. influence AA requirements [15]. Most of the earlier experiments on AA requirements of WL hens have been conducted for shorter duration. Here we have covered almost full laying cycle. Further there is a need to recommend the requirement of Lys for each production phase which vary in BW, EW and level of production. In this study we have attempted to estimate AA requirements for early, mid and late laying cycle separately to have more precise estimates. The interaction between dLys concentration and the period is clearly evident in present data. The effect of period represents the hen’s age. With advancing age, egg size increases, the interval between ovulation increases causing deposition of same amount of yolk in smaller number of follicles influencing weight and size of eggs [14]. Data on digestible AA requirements of WL hens for different laying phases under tropical condition is scanty. In the present study, it was assumed that dLys requirements will be between 0.700% to 0.563% during different phases of EP. The estimated requirements of dLys were 0.698%, 0.631%, and 0.586%, respectively during early, mid and late laying phases.

In early laying phase, based on the estimates of optimal dietary dLys concentrations and observed FI, daily intake of dLys was calculated to be 727 mg/b. In mid laying phase, daily intake requirements of dLys were similar to that of early laying phase. In late laying phase daily intake requirements were worked out to be 684 mg/b/d. The NRC [3] recommends a total Lys consumption of 690 mg/b/d to maximize EM, corresponding to 593 mg/b/d of true digestible Lys when applying a mean true Lys digestibility of 86% in corn and soybean meal based diet [3]. Bonekamp et al [16] and Spangler et al [17] reported that for Lohman LSL hens (light weight type), 600 to 620 mg/b/d dLys intake was sufficient for optimal laying percentage during early to mid laying phase. However, they observed that optimal Lys intake for maximum EW and FE were higher than the requirement for EP. Earlier, Rama Rao et al [18] estimated total Lys and Met requirements of WL layers (Babcock) from 21 to 72 wk of age in two trials involving supplementation of low CP (14.11% to 16.34% CP) high metabolizable energy (ME, 10.88 MJ/kg) diets with graded concentration of Lys (0.65% to 0.80%) and reported that these layers require 0.7% lysine in diets containing approximately 15% CP and 10.88 MJ of ME/kg but to achieve optimal EW a level of 16.5% CP is required. Our current estimates are considerably higher than those of our previous findings [18]. Similarly, the optimum concentration of dLys observed in the current study are considerably higher than the concentration reported by some other researchers [5 dLys, 526 to 561 mg/b/d) for layers of similar age. Similar to the data of the present experiment, few studies [19–21] have reported higher Lys (752 to 876 mg/b/d) requirement during the initial production phase. The relatively higher requirements of the dLys observed in the present study compared to some of the earlier reports may partly be attributed to higher rate of production (95% vs 83% to 89%) and also FI (106 g vs 88.7 g to 100 g/b/d) in the present study. The higher FI might have resulted in higher intake of these LAA in the current study compared to the reported values. The higher FI could be due to low energy concentration in diet of the present study (10.25 MJ/kg) compared to the dietary energy concentrations used by above authors (11.88 to 12.13 MJ/kg). The FI during the initial 2 periods was not affected by the AA concentration in diet. Another probable reason for higher dLys requirements suggested by Santos et al [20] and Pastore et al [21] could be due to strain variation (Isa Browen and Hy-Line W36) which are heavy in BW compared to the strain used in the current study.

The optimum requirement of Lys also depends on the protein content in the test diets as observed in our earlier studies [22]. Higher Lys (598 vs 584 mg/b/d) requirement was observed when the layers fed low protein diet (13.36% CP) compared to those fed optimum protein (15.78%).

The quantity of feed required to produce a unit egg reduced significantly with increase in dietary concentrations of dLys. Similarly, improvement in FE with increasing concentrations of Lys [17,23], Met, or total sulfur AA (TSAA) [24] in layers during post peak production was reported in the literature.

Majority of production parameters (EP, EW, and FE) were not affected by concentrations of dLys suggesting that the lowest level, i.e. 0.563% of dLys was adequate for these parameters. However, the FI was significantly reduced by dLys level. The daily intake requirements of dLys, during this phase works out to be 674 mg/b, respectively. These correspond to intake requirement of 783 mg/b/d total Lys when applying a mean true Lys digestibility of 86% in corn and soybean meal diet. The derived daily intake requirements of total Lys is similar to the values of total Lys (715 to 816 mg/b/d) requirements for layers reported by Novak et al [24] during mid to late laying phase. On the contrary, several authors have reported lower requirements of dLys [5, 561 to 526; 26, 538 to 561; 17, 600 mg/b/d) compared to the intake requirement observed in the current study. The variation in the AA requirement might be due to the differences in CP level tested by various authors. Lower concentration of Lys (561 vs 526 mg/b/d) requirements were reported for optimum EP [5] with reduction in dietary CP from 14.3% to 13.65%. Similarly, improvement in EP, EM, and FE were observed in layers (45 to 56 weeks of age) fed diets with 0.7% total lysine [6] with a calculated intake of 558 mg dLys/b/d which is considerably lower than the values observed in the current study. The lower EP (56.2%) observed in their study could be the reasons for the lower dLys requirements suggested by the authors.

Earlier, for the entire production or post peak phase (45 to 53 or 24 to 60 wk of age, respectively), Bonekamp et al [16] and Harms and Russell [25] did not observe any difference in EP by reducing concentrations of Lys (800 to 550 mg/b/d) in diet. The calculated mean daily intake of dLys in these studies was 600 mg/bird, which was marginally lower than the concentration observed in the present study (674 mg/b/d). The lower requirements suggested by these authors may be due to use of lower concentrations of AA in relation to the higher levels of ME (11.72 and 12.13 MJ/kg) in diet, which consequently reduced the average daily FI (110 and 88.7 g/b/d vs 115 g/b/d) and intake of the AA in their studies.

The BW was not affected by increasing concentration of LAA in diet, which is contrary to our previous report [18], wherein BW increased significantly with increase in dietary Lys from 0.65% to 0.70% in diet and further increase in Lys to 0.75% did not improve the BW indicating that the optimum requirement of total Lys for BW gain in WL layers is about 0.70%. The minimum level of dLys used in the present study (0.563%) correspond to total Lys level of 0.65% which is nearer to the optimum Lys concentration (0.70%) suggested in our earlier study [18]. Similar to the present findings, Sohail et al [26] did not observe any difference in BW in layers fed higher concentrations of SAA (0.65%, 0.72%, or 0.81%).

Considering the FI and performance data, the calculated daily intake of digestible TSAA works out to be 651 mg/b in the current study. Similarly, Rama Rao et al [18] estimated total TSAA requirements of WL layers (Babcock, Venkateswara Hatcheries Pvt Ltd, Pune, India) from 21 to 72 wk of age and found optimum EP and BW at an intake of 532 mg/b/d, which is considerably lower than the values found in the current study. The higher requirements observed in the current study could be due to inclusion of sunflower meal and rapeseed meal in addition to higher levels of deoiled rice bran in the test diets. In our previous study [18] soybean meal was the primary source of protein whose digestibility is known to be higher than the alternate feed ingredients [27]. Similar to these observations, our recent study [13] also found higher requirements of TSAA (575 to 681 mg/b/d) for WL layers fed diets with less digestible protein source (guar meal 10%) compared to those fed the soybean meal based diets (502 to 572 mg/b/d).

The EP realized in the current study was about 2% to 4% lower at different phases of production than the standard suggested (BV 300 layer management guide, Venkateswara Hatcheries Pvt Ltd, India). Similarly, the EW was about 2 to 5 g lower during the early phase of production but the difference reduced to 1 to 2 g in the late phase of EP. The lower production observed in the current study could be due to lower FI (1 to 6 g/b/d) than the suggested in the manual. However, the requirement of dLys in the current study was higher than the levels recommended for the strain tested.

The EW was not affected by the variation in concentrations of dLys tested in the present study. Contrary to these findings, few authors [16,25,28] have reported increased egg size with increases in intake or concentration of Lys, Met, Thr, or Try in diet. The variation in response of EW between the current experiment and that reported in literature may be due to the variation in concentration of the LAA. The above authors studied the response of supplementing a single AA in diets containing adequate concentration of other AA from deficit to high concentration. Supplementation of the most LAA in their studies might have improved EW. Some of these authors used diets containing unusual feed ingredients and supplemented the diet with essential AA in crystalline form to create deficiency of the specific AA studied. While in the current study, diets were prepared with practical feed ingredients and the concentration of Lys, Thr, and Try in the lowest dLys diets (0.563%, 0.464%, and 0.151%, respectively) were higher than the adequate level [3] without supplementing crystalline AA.

## CONCLUSION

The study indicated that WL layers require 727 mg/b/d of dLys during the initial (27 to 38 wk) and mid (39 to 58 wk) production phase, while during the late laying phase (59 to 74 wk) an intake of 684 mg/b/d was found optimum for EP, FE, FI, and EW when the diet contained 15% protein and about 10.25 MJ/kg ME.

## Figures and Tables

**Table 1:** Ingredient and nutrient composition of diets with different concentrations of dLys

Items	dLys (%)				

	0.700	0.665	0.630	0.595	0.563
Ingredient (g/kg)					
Maize	499.00	514.00	517.00	506.00	485.00
Deoiled rice bran, 15.5%	200.00	201.00	200.00	220.00	218.00
Soy bean meal, 46% CP	148.00	132.00	130.00	100.00	80.00
Sunflower cake, 28.5% CP	0	0	0	23.00	66.00
Rapeseed cake, 36% CP	40.00	40.00	40.00	40.00	40.00
Lime stone powder	20.00	20.00	20.00	20.00	20.00
Stone grit	80.00	80.00	80.00	80.00	80.00
Dicalcium phosphate	5.50	5.60	5.60	5.50	5.25
Salt	4.30	4.30	4.30	4.30	4.30
DL-methionine	1.47	1.29	0.98	0.62	0.22
L-lysine HCl	0.65	0.68	0.29	0.00	0.00
Premix^1)^	1.20	1.20	1.20	1.20	1.20
Phytase 5,000 (μ/g)	0.10	0.10	0.10	0.10	0.10
Nutrient composition (g/kg)					
Metabolizable energy (MJ/kg)^2)^	10.23	10.30	10.32	10.22	10.17
Protein^3)^	152.2	150.2	148.8	151.3	151.1
dLysine^3)^	7.00	6.65	6.30	5.95	5.63
dMethionine^4)^	3.83	3.59	3.28	2.97	2.66
dSAA^4)^	6.16	5.85	5.54	5.24	4.93
Calcium^2)^	35.0	35.0	35.0	35.0	35.0
Available phosphorus^2)^	2.8	2.8	2.8	2.8	2.8

dLys, digestible lysine; CP, crude protein; dSAA, digestible sulfur amino acids.

1) Provided (mg/kg diet): thiamin 1; pyridoxine, 2; cyanocobalamine, 0.01; niacin, 15; pantothenic acid, 10; a tocopherol, 10; riboflavin, 10; biotin, 0.08; menadione, 2; retinol acetate, 2.75; cholecalciferol, 0.06; choline, 650; copper, 8; iron, 45; manganese, 80; zinc, 60; selenium, 0.18; hydrated sodium calcium alumino silicates, 800; phytase, 375 units.

2) Calculated.

3) Analyzed.

4) Calculated based on analyzed ingredient composition.

**Table 2:** Effect of graded concentrations of digestible lysine on various performance parameters in White Leghorn layers during first phase of laying (27 to 38 wk)

P	Age (wk)	dLys (%)					SEM	Significance of effects (p-value)			Helmert contrasts for P	Polynomial contrasts for dLys
	
		0.563	0.595	0.630	0.665	0.700		dLys	P	P×dLys		
Egg production (%)												
1	27–30	93.6	93.6	94.2	95.1	96.0	0.465	0.03	<0.001	0.391	P1vs later: <0.001; P2 vs P3: <0.001	L: <0.001; Q: 0.889; C: 0.217; Order4: 0.890
2	31–34	94.3	94.1	95.0	95.9	96.8	0.534					
3	35–38	90.6	90.8	91.9	92.6	91.2	0.659					
Feed intake (g/b/d)												
1	27–30	105.2	106.7	106.4	106.3	106.1	0.395	0.102	<0.001	0.692	P1vs later: <0.001; P2 vs P3: <0.001	L: <0.001; Q: 0.889; C: 0.217; Order 4: 0.890
2	31–34	108.3	109.4	109.3	109.5	108.5	0.467					
3	35–38	105.0	106.1	105.0	106.2	104.4	0.695					
Feed efficiency (g food/egg)												
1	27–30	111.6	114	111.8	113.7	110.7	0.509	<0.001	<0.001	0.086	P1vs later: <0.001; P2 vs P3: 0.079	L: <0.027; Q: 0.014; C: 0.790; Order 4: <0.001
2	31–34	113.9	116.3	114	116.2	112.2	0.509					
3	35–38	116.0	116.9	114.3	114.8	114.6	0.867					
Egg weight (g/b/d)												
1	27–30	50.7	50.6	50.8	50.8	51.0	0.18	0.484	<0.001	0.462	P1vs later: <0.001; P2 vs P3: <0.001	L: 0.080; Q: 0.798; C: 0.877; Order 4: 0.663
2	31–34	53.9	53.7	53.7	53.9	54.0	0.107					
3	35–38	52.7	53	52.9	53.1	52.9	0.149					
Egg mass (g/d)												
1	27–30	47.8	47.4	48.4	47.5	48.9	0.332	0.013	<0.001	0.045	P1vs later: <0.001; P2 vs P3: <0.001	L: 0.004; Q: 0.527; C: 0.895; Order 4: 0.0332
2	31–34	51.2	50.6	51.4	50.8	52.2	0.294					
3	35–38	47.7	48.1	48.6	49.1	48.2	0.382					

P, period; dLys, digestible lysine; SEM, standard error of the mean.

**Table 3:** Effect of graded concentrations of digestible lysine on egg production, feed intake and feed efficiency in White Leghorn layers during second phase of laying (39 to 58 wk)

Items	Egg production (%)					Feed intake (g/b/d)					Feed efficiency (g food/egg)				
		
Period	1	2	3	4	5	1	2	3	4	5	1	2	3	4	5
Age (wk)	39–42	43–46	47–50	51–54	55–58	39–42	43–46	47–50	51–54	55–58	39–42	43–46	47–50	51–54	55–58
d Lys (%)															
0.563	88.0	91.8	88.6	87.9	87.5	110.2	115.5	117.8	123.8	120.6	125.1	125.8	133.0	140.9	137.8
0.595	88.7	92.3	90.9	89.8	87.4	111.8	116.5	119.3	125.6	121.6	124.0	126.2	133.2	139.9	139.0
0.630	90.1	91.9	90.4	89.8	88.0	107.0	114.2	115.0	122.9	118.7	120.6	124.2	127.3	136.9	134.8
0.665	91.0	93.9	92.5	91.7	89.9	111.4	117.3	118.1	126.3	123.7	122.4	124.9	127.6	137.7	137.7
0.700	89.1	92.1	89.1	88.2	87.9	107.5	112.5	114.0	120.2	117.6	120.6	122.2	127.9	136.4	133.9
SEM	0.605	0.698	0.875	0.998	0.874	1.31	1.05	1.21	1.26	1.32	1.11	0.982	1.52	1.49	1.40
Significance of effects (p-value)															
d Lys	0.021					0.007					0.002				
Period	<0.0001					<0.0001					<0.0001				
d Lys×Period	0.631					0.709					0.862				
Helmert contrasts for periods	P1vs later:0.050; P2 vs later:<0.001; P3 vs later:<0.001; P4 vs P5:0.001					P1vs later:<0.001; P2 vs later:<0.001; P3 vs later:<0.001; P4 vs P5:<0.001					P1vs later:<0.001; P2 vs later:<0.001; P3 vs later:<0.001; P4 vs P5:0.053				
Polynomial contrasts for d Lys	L0.156;Q:0.024;C:0.097; Order 4:0.126					L:0.079;Q:0.165;C:0.228; Order 4:0.003					L:<0.001;Q:0.564;C:0.923; Order 4:0.035				

dLys, digestible lysine; FE, feed efficiency; SEM, standard error of the mean.

**Table 4:** Effect of graded concentrations of digestible lysine on egg weight in White Leghorn laying hens during second phase of laying (39 to 58 wk)

Items	Egg weight (g/b/d)					Egg mass (g/b/d)				
	
P	1	2	3	4	5	1	2	3	4	5
Age (wk)	39–42	43–46	47–50	51–54	55–58	39–42	43–46	47–50	51–54	55–58
dLys (%)										
0.563	53.6	54.9	55.5	57.6	57.8	47.2	50.4	49.2	50.7	50.6
0.595	53.5	55.1	55.9	57.5	57.9	48.2	50.9	50.8	51.6	50.6
0.630	53.6	54.9	55.8	57.6	57.9	47.6	50.5	50.5	51.7	50.9
0.665	53.7	55.0	55.5	57.7	57.5	48.9	51.7	51.4	52.9	51.7
0.700	53.5	55.0	55.6	57.5	57.7	47.7	50.7	49.5	50.8	50.7
SEM	0.147	0.082	0.141	0.124	0.326	0.372	0.379	0.486	0.585	0.621
Significance of effects (p-value)										
dLys	0.925					0.034				
P	<0.001					<0.001				
dLys×P	0.863					0.639				
HC for P	P1 vs later: <0.001; P2 vs later: <0.001; P3 vs later: <0.001; P4 vs P5: 0.241					P1 vs later: <0.001; P2 vs later: 0.558; P3 vs later: <0.001; P4 vs P5: 0.010				
PC for dLys	L: 0.797; Q: 0.443; C: 0.652; Order 4: 0.992					L: 0.249; Q: 0.032; C: 0.221; Order 4: 0.063				

dLys, digestible lysine; P, period; SEM, standard error of the mean; HC, Helmert contrast; PC, polynomial contrast.

**Table 5:** Effect of graded concentrations of digestible lysine on performance parameters in White Leghorn laying hens during third phase of laying (59 to 74 wk)

P^1)^	dLys (%)					SEM	Significance of effects (p-value)			Helmert contrasts for P	Polynomial contrasts for dLys
	
	0.563	0.595	0.630	0.665	0.700		dLys	P	P×dLys		
Egg production (%)											
1	84.8	84.4	85.5	86	84.2	1.06	0.694	<0.001	0.418	P1 vs later: <0.001; P2 vs later: <0.001; P3 vs P4: <0.001	L: 0.686; Q: 0.205; C: 0.555; O4: 0.902
2	83.0	82.0	84.2	83.2	83.0	1.09					
3	81.1	81.6	81.9	82.6	81.6	0.456					
4	77.0	77.5	77.9	78.5	77.5	0.433					
Feed intake (g/b/d)											
1	114.5	118	113.8	118.4	111.6	1.40	0.038	<0.001	0.138	P1 vs later:0.362; P2 vs later: <0.001; P3 vs P4: <0.001	L: 0.230; Q: 0.080; C: 0.355; O 4: 0.017
2	111.2	111.1	111.3	111.6	110	1.45					
3	115.5	116.9	114.6	117.2	113.5	0.761					
4	117.9	119.3	117	119.6	115.8	0.776					
Feed efficiency (g food/egg)											
1	135.3	136.6	133.2	137.7	132.6	3.13	0.100	<0.001	0.791	P1 vs later 0.524; P2 vs later: 0.871; P3 vs P4: 0.006	L: 0.105; Q: 0.581; C: 0.733; O 4: 0.025
2	134	135.6	132.3	134.4	132.6	1.75					
3	142.5	143.3	139.9	141.9	139.2	0.818					
4	153.1	154	150.3	152.5	149.5	0.879					
Egg weight (g/b/d)											
1	57.8	58.1	58	58.5	58.2	0.383	0.354	<0.001	0.852	P1 vs later: 0.015; P2 vs later: <0.001; P3 vs P4: <0.001	L: 0.371; Q: 0.160; C: 0.718; O 4: 0.239
2	56.4	56.7	56.6	56.8	56.3	0.161					
3	58.0	58.1	58.1	58.2	58.1	0.075					
4	60.9	61.0	61.0	61.1	61.0	0.079					
Egg mass (g/d)											
1	49.0	50.2	49.6	50.3	49.0	0.677	0.425	<0.001	0.607	P1 vs later: <0.001; P2 vs later: 0.072; P3 vs P4: <0.001	L: 0.522; Q:0.104;C:0.495; O 4:0.641
2	46.8	46.6	47.7	47.2	46.7	0.628					
3	47.0	47.4	47.6	48.0	47.4	0.267					
4	46.9	47.3	47.5	47.9	47.2	0.285					

dLys, digestible lysine; SEM, standard error of the mean; L, linear; Q, quadratic; C, cubic; O4, order 4.

1) P, period (1, 59 to 62 wks; 2, 63 to 66 wks; 3, 67 to 70 wks; 4, 71 to 74 wks).

**Table 6:** Requirements of dLys for laying hens fed diet containing graded concentrations of dLys during different phases

Response variable	Model	Equation	Estimated requirement (100%, 95%)	Sum of square of residuals	p-value	R^2^
Phase1 (27 to 38 wk)						
Egg production	E	Y = 75.44+26.12×[1−EXP(−1.907×X)]	0.858, 0.815	40.9	<0.01	0.329
Egg mass	QBL	Y = 49.3–28.74×(0.695−X)^2^	0.695, 0.660	14.25	<0.01	0.212
Feed efficiency	QP	Y = 18.56+314.6–257.2X^2^	0.612, 0.581	87.4	<0.01	0.179
Phase 2 (39 to 58 wk)						
Egg production	QP	Y = −52.2+444.1X–344.7X^2^	0.644, 0.612	122.5	<0.01	0.171
Egg mass	QBL	Y = 51.1–79.85× (0.700–X)^2^	0.700, 0.665	41.8	< 0.01	0.201
Feed efficiency	QP	Y = 202.2–197.9X+132.4X^2^	0.747,0.709	211.8	<0.01	0.295
Feed intake	QP	Y = 8.29+364.1X–301.8X^2^	0.603, 0.573	386.6	<0.01	0.092
Phase 3 (59 to 74 wk)						
Feed intake	QP	Y = −27.3+463.9X–375.7X^2^	0.617,0.586	287.0	<0.01	0.092

dLys, digestible lysine; E, exponential; QP, quadratic polynomial; QBL, quadratic broken line.
